# The Panel of 12 Cell-Free MicroRNAs as Potential Biomarkers in Prostate Neoplasms

**DOI:** 10.3390/diagnostics10010038

**Published:** 2020-01-10

**Authors:** Maria Yu. Konoshenko, Evgeniy A. Lekchnov, Olga E. Bryzgunova, Ivan A. Zaporozhchenko, Sergey V. Yarmoschuk, Oksana A. Pashkovskaya, Svetlana V. Pak, Pavel P. Laktionov

**Affiliations:** 1 Institute of Chemical Biology and Fundamental Medicine SB RAS, 630090 Novosibirsk, Russia; 2 E.N. Meshalkin National Medical Research Center of the Ministry of Health of the Russian Federation, 630055 Novosibirsk, Russia

**Keywords:** prostate cancer, miRNA, urine, extracellular vesicles

## Abstract

Prostate cancer is a global biological, medical, and social issue aggravated by the lack of reliable, highly specific, and sensitive non-invasive tests for diagnosis and staging of prostate cancer. One prospective source of biomarkers are the cell-free miRNAs present in various biological fluids. In the present study, we validated the diagnostic potential of cell-free miRNAs: miR-19b, miR-22, miR-92a, miR-378, miR-425, miR-30e, miR-31, miR-125b, miR-200b, miR-205, miR-375, and miR-660; we estimated the required sample size and the minimal miRNA set for a subsequent large-scale validation study. Relative expression of 12 miRNA combined in 31 ratios was investigated in three fractions of biological fluids (urine extracellular vesicles, clarified urine, and plasma) obtained from patients with prostate cancer (n = 10), benign prostate hyperplasia (n = 8), and healthy volunteers (n = 11). Eight of the miRNAs found in urine vesicles (miR-19b, miR-30e, miR-31, miR-92a, miR-125, miR-200, miR-205, and miR-660) showed great promise and when combined into six ratios (miR-125b/miR-30e, miR-200/miR-30e, miR-205/miR-30e, miR-31/miR-30e, miR-660/miR-30e, and miR-19b/miR-92a) could classify patients with prostate cancer, benign prostate hyperplasia, and healthy donors with 100% specificity, 100% sensitivity, and with a high degree of reliability for most donors.

## 1. Introduction

Prostate cancer (PCa) is the second most common cancer with a predicted further increase in incidence over the following years and the fifth leading cause of cancer-related deaths in men, thus presenting a significant biological, medical, and social problem [1,2]. Serum prostate-specific antigen (PSA), digital rectal examination (DRE), and transrectal ultrasound (TRUS) followed by ultrasound-guided biopsy of the prostate constitute the foundation of modern clinical diagnosis for PCa. However, the sensitivity and the specificity of these methods are insufficient for effective detection and monitoring of PCa [3]. Moreover, the United States Preventive Services Task Force (USPSTF) has repeatedly issued recommendations against widespread use of PSA as a screening marker due to overdiagnosis and uncertain impact on patients’ survival. Despite the contradicting view of the European Association of Urology (uroweb.org/guideline/prostate-cancer/#5), low specificity of PSA calls for a reliable, highly specific, and sensitive alternative for diagnosis and staging of PCa. There is a special demand for low cost, simple, and minimally invasive screening tests to facilitate early detection and tumor monitoring after therapy without added health risks and overdiagnosis. Liquid biopsy based on detection of cell-free miRNA is a fast-evolving field with the potential to be successfully used in clinical cancer diagnostics. Many miRNAs are known to be involved in oncogenesis, tumor progression, and metastasis by targeting tumor suppressors, oncogenes, or other proteins associated with disease progression or drug resistance [4,5,6,7,8]. Differential expression of miRNAs in biological fluids of patients with different cancers and healthy donors has been previously reported by many groups [7,9,10,11,12,13,14,15]. Other reports also showed extracellular vesicles (EVs) and cell-free nucleoprotein complexes carry distinct populations of miRNA, and the content of these miRNA pools can be used to develop promising tools for PCa screening [16,17]. We recently analyzed the expression of 84 miRNAs in paired samples of urine EVs and cell-free urine supernatant from healthy donors as well as patients with benign and malignant prostate tumors and revealed sets of miRNAs differentially expressed between PCa and control groups [3]. Based on these results, we designed an algorithm comprising 17 analytical systems that allowed for PCa detection with 97.5% accuracy [18].

Here, we aim to validate candidate biomarker miRNAs selected from our previous data [3,18] in blood plasma, clarified urine, and urine EVs by quantitative reverse transcription PCR (qRT-PCR) in order to define the minimal set of miRNA markers and estimate the required sample size for subsequent large-scale validation study.

## 2. Results

Based on previous data obtained by profiling of miRNA expression using LNA-based RT-PCR arrays (miRCURY LNA miRNA qPCR, Exiqon, Denmark) in paired samples of urine EVs and cell-free urine supernatant, we identified 12 candidate miRNA biomarkers of PCa [3,18]. For all miRNAs, qRT-PCR assays with a working range of 24–38 threshold cycles (Ct) of PCR were designed. Non-template controls produced no signal or were at least seven cycles away from the minimum detectable amount of specific template. All reported data were obtained using RNA samples that produced Ct values within the working range of the systems. Spike-in control (cel-miR-39) was detected in all samples at 25 ± 1 Ct.

Table 1 and Table 2 demonstrate the results of comparative expression analysis for miRNA ratios based on the difference of Ct difference (dCt) values (ddCt) or dddCt values in the respective categories. Only statistically significant differences are shown. There were no significant intergroup differences of miRNA ratios in blood plasma. Expression of 15 miRNA ratios (12 miRNA) in at least one fraction of the included biological fluids allowed differentiating between healthy men and PCa patients [Table 2.1; PCa vs. healthy male donors (HD)]. Among these ratios, 14 were detected in urine EVs and one in urine supernatant. Levels of 20 miRNA ratios (10 miRNAs) could differentiate between PCa and benign prostatic hyperplasia (BPH) patients. These miRNA ratios were found in urine EVs (14 ratios) and urine supernatant (nine ratios). Of particular interest were the 10 miRNA ratios for which dCt values for HD and BPH groups were both located above or below those for PCa patients (i.e., dCt values had the same sign). This common directionality of differences in PCa comparisons with HD and BPH suggests they can potentially differentiate malignant from benign or absence of disease (Table 1, highlighted in yellow). Finally, 15 miRNA ratios were different between BPH patients and healthy volunteers, including 15 ratios from urine EVs and two from urine supernatant. For five miRNA ratios, the difference between HD and PCa or BPH had the same direction (Table 1, highlighted by bold). Considering statistical distribution of the data, the minimal sample size to confirm these differences at 95% significance and power did not exceed 18 participants per group, increasing to 35 per group at 99% significance.

Study of miRNA representation revealed 20 miRNA ratios with significant differences in ddCt values for any two sample types between PCa patents and healthy men (Table 2), including 16 ratios differently distributed between urine EVs and supernatant, and one and 15 ratios for comparisons of urine EVs and urine supernatant with plasma, respectively. Similarly, 15 miRNA ratios were differently distributed between PCa and BPH patients (13 for urine EVs–urine supernatant, none for urine supernatant-blood plasma, 11 for urine EVs–blood plasma). Common directionality of differences in PCa comparisons with HD and BPH was found for 21 dddCt values (Table 2, highlighted by bold). Distribution of nine miRNA ratios for urine EVs–blood plasma was significantly different between BPH patients and healthy men patients. Twenty-one miRNA ratios for PCa–HD and PCa–BPH comparisons had the same sign of the difference in distribution, while for HD–PCa and HD–BPH comparisons, the number of ratios with identical signs was only seven. Two miRNA ratios were differently distributed between all three groups in a progressive manner—miR-miR-31/miR-30e, and miR-200b/miR-30e for urine EVs and blood plasma (Table 2). Notably, the selection of differently distributed ratios was not identical to differently expressed miRNA ratios in any of the sample types.

Minimal sample size required for verification of these data (Table 2) was no more than 35 at 95% significance and power, and 40 per group at 99% significance (with the exception of miR-660/miR-375 ratio, which would require 75 participants per group).

The receiving operator characteristic (ROC) curve analysis was used to measure the diagnostic performance of miRNA ratios in the donor classification. Table 3 and Table 4 show sensitivity at 100% specificity for discrimination of PCa patients from control group (BPH+HD) and pairwise classification of PCa from HD, PCa from BPH, and BPH from HD, respectively.

**Figure 1:**
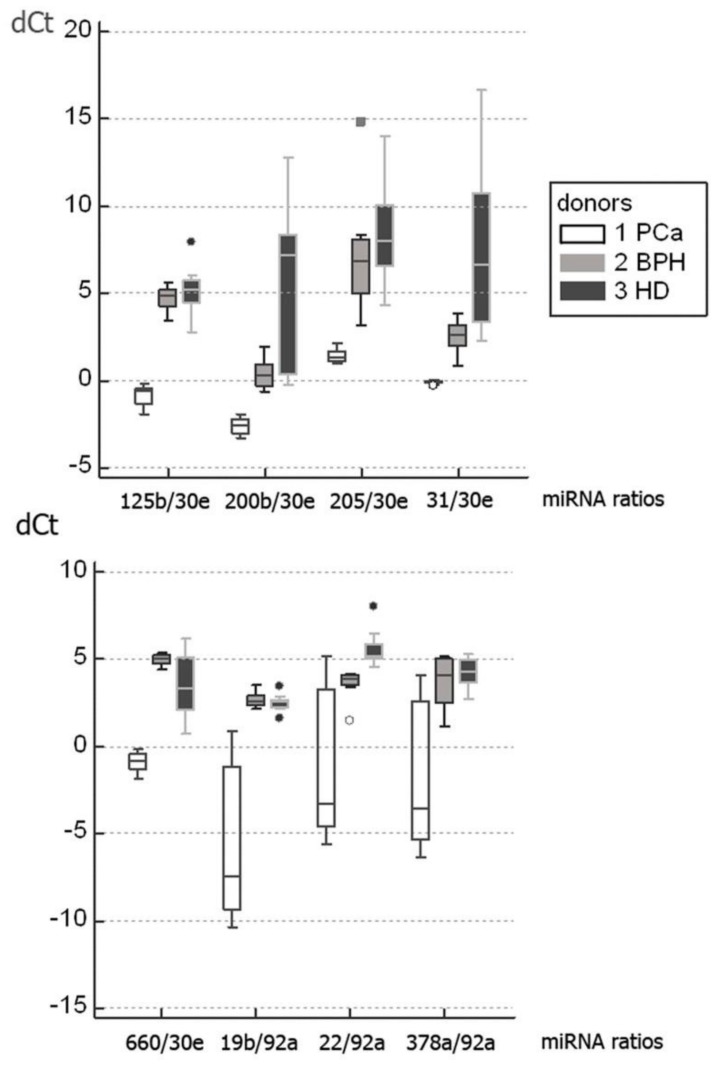
The dCt values of miRNA ratios isolated from urine EVs with the highest diagnostic value.

The miRNAs isolated from urine EVs demonstrated the highest diagnostic value (Table 3, Table 4 and Table 5) with six miRNA ratios (miR-125/miR-30e, miR-200/miR-30e, miR-205/miR-30e, miR-31/miR-30e, miR-660/miR-30e, and miR-19b/miR-92a) allowing discrimination of PCa patients from the combined control group of healthy donors and BPH patients with 100% sensitivity and 100% specificity (Table 3). Figure 1 demonstrates the dCt values for these miRNA ratios.

Four additional ratios could discriminate PCa patients and the control group with sensitivity between 80% and 100% at absolute specificity: miR-375/miR-30e, miR-22/miR-19b, miR-378a/miR-19b, and miR-425/miR-92a [area under the curve (AUC) = 0.90, 0.86, 0.98, 0.99, respectively] (Table 3). Analysis of distribution between different fractions could increase the sensitivity of classification, as shown for miR-22/miR-19, miR-378/miR-19, and miR-425/miR-92 (Table 3). In several cases, classification made on the basis of miRNA ratio distribution achieved absolute sensitivity and specificity—for example, in miR-30/31, miR-19/92, and miR-125/30—however, in all of these cases, expression of miRNA ratio in urine EVs alone yielded the same classification efficiency (Table 3).

Discrimination of PCa patients and healthy men with 100% sensitivity and 100% specificity could be achieved with miR-125/miR-30, miR-200/miR-30, miR-205/miR-30, miR-31/miR-30, and miR-19/miR-92 in urine EVs and miR-22/miR-92 for ddCt urine EVs–blood plasma (Table 4). Three more miRNA pairs discriminated PCa and HD with 100% specificity and sensitivity 80% or 90%: miR-378/miR-19, miR-425/miR-92 (AUC = 0.97, 0.99, respectively; urine EVs), and miR-22/miR-19 (AUC = 0.92, ddCt urine supernatant–blood plasma) (Table 4).

The following miRNA ratios were effective in the discriminating PCa from BPH: miR-125/miR-30, miR-125/miR-31, miR-200/miR-125, miR-200/miR-30, miR-205/miR-30, miR-31/miR-30, miR-660/miR-200, miR-660/miR-30, miR-660/miR-31, miR-19/miR-92, miR-22/miR-19, miR-378/miR-19, and miR-425/miR-19 in urine EVs and miR-375/miR-200 in ddCt urine EVs–blood plasma with 100% sensitivity and 100% specificity (Table 5). Five additional ratios achieved lower sensitivity (80–100%) in classifying PCa and BPH patients: miR-22/miR-425 (AUC = 0.91; urine EVs), miR-425/miR-92 (AUC = 0.94, ddCt urine EVs–blood plasma) miR-375/miR-30, miR-660/miR-375, and miR-660/miR-125 (AUC = 0.84, 0.98, 0.89, respectively; ddCt urine EVs–urine supernatant) (Table 5).

Only the miR-22/miR-92a ratio measured in urine EVs could discriminate BPH patients from healthy men with 100% sensitivity and 100% specificity (Table 6). Three ratios demonstrated lower sensitivity (80–100%): miR-125/miR-30e, miR-200/miR-125 (AUC = 0.89; 0.91; urine supernatant), and miR-200/miR-30e (AUC = 0.99, ddCt urine EVs–blood plasma) (Table 6).

To select the most diagnostically efficient miRNA pairs, we used a modification of the algorithm described earlier [18]. Here, we followed only the first two steps for miRNAs ratios with the highest sensitivity in previous assays (50% or higher sensitivity of discrimination between case and control groups) to obtain an overlapping diagnostic system with maximum stability:

**Step 1.** Data are divided into case and control groups. If normality of distribution is confirmed, mean and standard deviation (SD) for the control group are calculated; if normality is rejected, median, 5% and 95% quantiles (Q5, Q95) are calculated instead. This is done for all candidate biomarkers.

**Step 2.** Set cut-off values to [mean + 2SD] or [mean − 2SD] calculated from control group. If the mean value in the case group is smaller than in the control group, −2SD is used, otherwise +2SD is used. Patients with values above [mean + 2SD] or below [mean − 2SD] are considered positive for tested condition based on that biomarker.

Here, normality was confirmed for all candidates, thus mean and SD values were used to generate cut-offs. Table 7, Table 8, Table 9 and Table 10 show the percentage of cases correctly classified from the control group by every miRNA ratio using this approach. Each of the PCa patients could be discriminated from the control group (HD+BPH) based on 7–21 dCt or ddCt of miRNA ratios. In total, 11 miRNAs could be used to separate these groups (Table 7).

In the reverse situation, each of the donors in the combined non-cancer group (HD+BPH) was discriminated from the control group (PCa patients) based on 14–23 dCt or ddCt of miRNA ratios. To discriminate the groups in such a manner, 12 miRNAs were used (Table 8).

All PCa patients and all but one BPH patient were discriminated from healthy donors based on 2–14 dCt or ddCt of miRNA ratios. The remaining BPH patient could only be classified using ddCt of miR-22/miR-92a between urine EVs and blood plasma. To completely separate these groups, 10 miRNAs were used (Table 9).

In the final assay, healthy donors could be discriminated from the control group of PCa and BPH patients based on 5–9 dCt or ddCt of miRNA ratios. One healthy donor could not be correctly classified by any of the ratios in any sample type. The inclusion of miR-375/miR-30e ddCt between urine EVs and blood plasma allowed discriminating this donor correctly. A total of six miRNAs were required to discriminate the groups (Table 10).

Correlation of miRNA ratio expression levels with clinical and demographic parameters [tumor size (TNM), Gleason score, blood PSA, and age] was examined. No correlations (k >= 0.6) between donor characteristics and miRNA ratio levels in urine supernatant or blood plasma were found. Strong correlation between levels of miR-19b/miR-92a (k = −0.76), miR-22/miR-92a (k = −0.73), miR-378/miR-92a (k = −0.76), miR-425/miR-92a (k = −0.77), miR-30e/miR-125b (k = 0.78), and miR-205/miR-30e (k = −0.70) in urine EVs and PSA concentration was revealed. These correlations were mostly confounded by the fact that blood PSA was used as one of the criteria when forming the groups. As such, PCa and BPH patients had elevated PSA values, which is not necessarily the case in the general population. Tumor size (T) positively correlated with miR-205/miR-200b ratio in urine EVs (k = 0.79). No meaningful correlation with Gleason’s score was identified for any of the ratios. Notably, none of the miRNA ratios correlated with donor age.

## 3. Discussion

Here, we investigated relative expression levels of 12 miRNAs assembled into 31 ratios in three fractions of biological fluids (urine EVs, urine supernatant, and plasma) from patients with PCa, BPH, and healthy men. With the exception of miR-378/miR-425, miR-200b/miR-31, miR-205/miR-125b, miR-205/miR-375, and miR-205/miR-660, all ratios were differently expressed between at least one pair of groups in at least one of the fractions (Table 1 and Table 2). Urine EVs were the source of the majority of differentially expressed miRNA ratios. This is in line with the latest trend suggesting EVs as a potent source of biomarkers, such as PCA3 and TMPRSS2:ERG fusion transcripts ([19], for review), including the test widely available in the US, the ExoDx Prostate Intelliscore test, which comprises PCA3, TMPRSS2:ERG, and SPDEF [20]. Alterations of miRNA distribution between urine EVs, supernatant, and plasma were also discovered and were shown to correlate with prostate health (Table 2). This additional data could in several cases increase the diagnostic sensitivity of the system, as illustrated by the distribution of miR-22/miR-19 and miR-378/miR-19 (ddCt, urine EVs, and urine supernatant) (Table 3). The results obtained are consistent with our previous reports [3,18].

One major difficulty in PCa diagnostics is discriminating malignant and benign prostate tumors. Pca and BPH foci can co-exist in the same patient, and the difference in miRNA expression in prostate tissue and biological fluids of PCa and BPH patients is generally far less pronounced than it is for PCa patients and healthy men [18]. Here, we identified 20 miRNA ratios that were differently expressed between PCa and BPH in at least one sample type, 14 of which could differentiate PCa and BPH patients with 100% sensitivity and specificity (Table 9). Particular consideration should be given to miRNA ratios that can reliably distinguish PCa from both BPH and HD (Table 7). These results show great promise in tackling the issue of differential diagnosis of prostate lesions, but they require additional verification in a larger sample to confirm the performance of miRNA ratios and select the best biomarker candidates.

We found meaningful correlation between blood PSA and relative expression of miR-19b/miR-92a, miR-22/miR-92a, miR-378/miR-92a, miR-425/miR-92a, miR-30e/miR-125b, and miR-205/miR-30e in urine EVs. These ratios were very effective in classifying PCa patients using a diagnostic algorithm (Table 7). This can be viewed as a confirmation of their diagnostic potential but is in fact a limitation of their use alongside PSA. However, their use alongside PSA should be further studied in non-PCA-preselected groups. Another five miRNA ratios (miR-200/miR-30e, miR-205/miR-30e, miR-31/miR-30e, miR-660/miR-30e, and miR-22/miR-378a) were not associated with PSA level while achieving 100% sensitivity and 100% specificity of PCa detection. These ratios may prove to be more suitable candidates for diagnostic routines combining newly developed markers with established clinical diagnostic tools.

We also discovered a positive correlation between tumor size (TNM) and miR-205/miR-200b ratio, but none of the miRNA ratios were related to Gleason score. This is partly consistent with an earlier report by Schaefer et al. [21] showing association of miRs-125b, -205, and -222 expression with tumor stage and significant correlation between Gleason score and miR−205 expression. This may indicate a role of miR-205 and miR-200b in tumor progression [22]. It is worth mentioning that our sample consisted of early stage PCa patients (T1-2, Gleason score 5–7), which benefits the search for biomarkers for differential diagnosis but restricts the clinical utility of selected markers for other applications, for example, tracking tumor progression and risk stratification. This may explain the overall lack of correlation between miRNA expression and clinical characteristics of patients. Due to the characteristics of the donors included in this study, it is not possible to differentiate low-risk and high-risk prostate cancer. However, it is an important goal of modern diagnostics and an ambitious aim of future studies.

Although there were inter-group differences of donors age (healthy 50–60 years old, PCa patients 54–71 years old, and BPH patients 63–81 years old, we also found no meaningful association between miRNA ratios and age. This is important because prostate tissue exhibits many age-related changes, which are often erroneously identified as neoplasms by other diagnostic tests, such as PCa, resulting in needless additional tests and false positive diagnoses. Absence of correlation between miRNA expression and age further supports the potential of selected ratios for PCa diagnosis.

Differential expression of miR-19b, miR-30e, miR-92a, miR-125b, miR-200b, miR-205, miR-378a, and miR-660 between PCa and healthy donors in biological fluids and/or tissues was demonstrated previously ([23,24,25,26,27,28,29,30,31,32,33,34,35,36,37,38] and others). These miRNAs are also known to be associated with specific aspects of PCa oncogenesis, including androgen receptor (AR) signaling [39,40], cell cycle regulation [40], cell proliferation and differentiation [26,41], epithelial–mesenchymal transition (EMT) [21,42,43,44], cell growth, apoptosis [45,46,47], adhesion and invasion [22], extraprostatic extension of the tumors [32], metastasis [38,42], biochemical failure [36,41], and hormone refractory [30,33]. Most of the identified miRNAs have known targets involved in PCa oncogenesis. All of these miRNAs have targets from cancer-related pathways such as TGF-β, FoxO, p53, ErbB, TNF, HIF, MAPK, and Wnt, and 11 of the miRNAs target the mTOR signaling pathway (DIANA-mirPath v.3, http://snf-515788.vm.okeanos.grnet.gr/). For example, miR-125b is involved in AKT/mTOR pathway regulation and targets candidate genes such as BAK1 and EIF4EBP1 associated with the AKT/mTOR pathway [21,27]. MiR-19b directly targets PTEN and TP53 and consequently reduces levels of their downstream targets such as Bax and p21 [48]. MiR-378 inhibits PCa development, reducing MAPK1 expression [27,31]. Down regulation of miR-200 in PC3 cells triggered by the growth factor PDGFD results in up-regulation of the transcription repressors ZEB1, ZEB2, and SNAI2, which regulate the loss of epithelial markers—a characteristic process in the epithelial–mesenchymal transition [22]. Presence of miR-205 is an essential factor for the inhibitory effects of p63, a metastasis suppressor, on EMT markers, ZEB1, and vimentin in PCa cells [42]. MiRNA-660 targets PCa-associated genes AMACR and PSMA [37]. Involvement in oncogenesis indirectly supports biomarker status of selected miRNAs and suggests they may have a broader clinical utility. Moreover, selected miRNAs regulate various signaling pathways that guarantee higher sensitivity and stability of the diagnostic panel. Previously, some researchers also attempted to create a diagnostic panel for the differential diagnosis of PCa and BPH patients based on the analysis of three (miR222-3p * miR-24-3p/miR-30c-5p) siRNAs (after checking 45 siRNA candidates) in clarified urine [49] and two siRNAs (miR 100/200b) in cell urine sediment [50]. The authors showed that such tests can be useful as an adjunct to PSA [in patients with PSA levels in the “gray area” (4–10 ng/mL)] to confirm the need for a prostate biopsy, but they cannot be used as stand-alone tests for diagnosing PCa. Despite the amount of reports describing the involvement of various miRNAs in PCa development, our results are among the few that demonstrate the potential of EVs-associated miRNAs as PCa biomarkers using a stable and robust ratio-based approach. For example, diagnostical potential of let-7c, miR-1, and miR-21 и miR-375 was shown by Laura Foj and coauthors [51].

**Figure 2:**
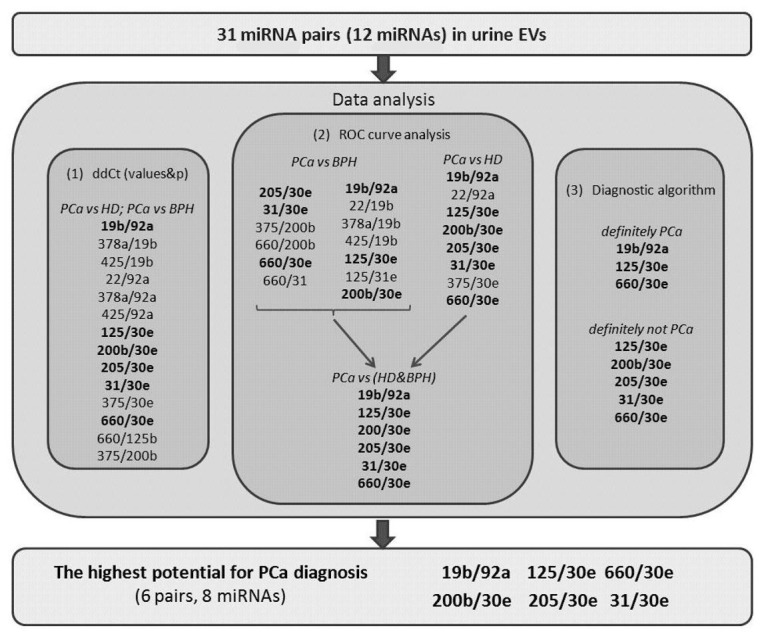
The course of the study.

Here, we used several approaches to analyze the data on miRNA expression and to select the most suitable miRNA ratios for PCa diagnosis (Figure 2). Figure 2 illustrates the course of the study and which miRNA ratios were the best according to each part of data analysis. According to our data, the most perspective miRNA ratios for PCa diagnosis were miR-125b/miR-30e, miR-200/miR-30e, miR-205/miR-30e, miR-31/miR-30e, miR-660/miR-30e, and miR-19b/miR-92a. These ratios were able to distinguish PCa patients with 100% sensitivity and 100% specificity and could be complemented by four other miRNAs (miR-375, miR-378, miR-22, and miR-425) to create four additional ratios with lesser sensitivity (80–90%). The analysis of the diagnostic algorithm, which includes these miRNA ratios, revealed that the most stable for distinguishing PCa patients from control group (HD+BPH) are miR-125b/miR-30e, miR-660/miR-30e, and miR-19b/miR-92a.

Thus, the most promising direction for a further verification with larger samples is the study of miR-19b, miR-30e, miR-31, miR-92a, miR-125, miR-200, miR-205, and miR-660 in urine EVs from PCa and BPH patients. At least 35 participants should be included in each group to achieve the desired statistical significance and power. Additionally, to facilitate clinical application of such biomarker panels, a simple and robust method for isolation of urine EVs should be developed and thoroughly characterized, as different EVs isolation techniques have shown to be selective towards different vesicle populations.

## 4. Materials and Methods

### 4.1. Sample Collection

Blood and urine samples from 11 healthy male donors (HD), 10 PCa, and 8 patients with benign prostatic hyperplasia (BPH) were obtained from E. Meshalkin National medical research center of the Ministry of Health of the Russian Federation (Novosibirsk, Russia). The age range and mean age, the blood PSA, the disease stage, and the Gleason score (for PCa patients) of the study population are shown by the Table 11. The study was approved by the ethics committee. Written informed consent was provided by all participants.

Venous blood was collected in EDTA spray-coated vacutainers, stored at 4 °C, and processed within 4 h. To obtain blood plasma, samples were sequentially centrifuged at 400× *g* for 20 min and at 800× *g* for 20 min, both at 4 °C. To remove cellular debris, samples were centrifuged at 17,000× *g* at 4 °C for 20 min.

Fresh urine samples were collected in sterile containers. Urinary cells and debris were removed by sequential centrifugation at 400× *g* for 20 min at room temperature and clarified at 17,000× *g* for 20 min at 24 °C to obtain urine supernatant.

### 4.2. Isolation of Urine EVs by Ultracentrifugation

Human urine (5 mL) was brought to 12 mL with phosphate-buffered saline (PBS), transferred to a 14 mL open top Ultra-Clear^TM^ centrifuge tube (Beckman Coulter, Brea, CA, USA), and centrifuged at 100,000 *g* for 90 min at 18 °C in a Beckman Coulter Optima TM L-90k centrifuge with SW 40Ti rotor (Beckman Coulter). The pellet was washed by resuspending in 10 mL of PBS and centrifugation in the same conditions. Finally, the pellet was resuspended in 500 µL PBS snap-frozen in liquid nitrogen and stored at −80 °C.

### 4.3. Isolation of miRNA by Gu/OcA Protocol

Before isolation of miRNA, blood plasma or urine samples were thawed and gently mixed. Gu/OcA miRNA isolation from urine and blood plasma was performed as described previously by Lekchnov et al. [52]. Isolation from urine EVs was performed as described for clarified urine. After the addition of denaturation buffer, synthetic cel-miR-39-3p was spiked-in the samples at 5 × 10^7^ copies per isolation.

### 4.4. RNA Precipitation

RNA precipitation by isopropanol was performed as described previously in Lekchnov et al. [52]. To stabilize the miRNA, 1.5 µL of glycogen (20 mg/mL) was added into each tube. Air dried miRNA pellets were dissolved in 30 µL of RNAse-free water.

### 4.5. Reverse Transcription and Quantitative RT-PCR

Reverse transcription (RT) on miRNA templates was performed as described by Chen et al. [53]. Primers and probes for reverse transcription and TaqMan qPCR (view Supplementary Materials) were synthesized in the Laboratory of Medicinal Chemistry (ICBFM SB RAS, Novosibirsk). Each RT reaction was performed in a total volume of 10 µL and contained 2.5 µL of RNA, 25 nM each of miRNA-specific primers, 0.5 unit of RiboLock RNAse inhibitor (Fermentas, Vilnius, Lithuania), 50 units of M-MuLV-RH reverse transcriptase (Biolabmix, Novosibirsk, Russia), 2 µL of 5× MMLV reaction buffer [250 mM Tris-HCl (pH 8.3 at 25 °C), 250 mM KCl, 20 mM MgCl_2_, 50 mM DTT], and 125 mM of each dNTP. The reaction conditions were as follows: 16 °C for 30 min, 42 °C for 30 min, and 70 °C for 10 min. Samples without RNA templates were used as negative controls.

Real-time PCR was carried out on the CFX 96TM Real-Time System (Bio-Rad, USA). All reactions were carried out in duplicate in a total volume of 24 µL. Each reaction contained 4 µL of RT product, 1 unit of Taq DNA polymerase (BiolabMix, Russia), 2.4 µL of 10× PCR buffer [750 mM TrisHCl (pH 8.8 at 25 °C), 200 mM (NH_4_)_2_SO_4_, 0.1% (*v*/*v*) Tween 20], 3.2 mM MgCl_2_, 200 mM of each dNTP, 480 nM miRNA-specific forward primer, 640 nM universal reverse primer, and 240 nM specific TaqMan probe (see Supplementary Materials, Table 11). After an initial denaturation at 95 °C for 3 min, the reactions were run for 50 cycles at 95 °C for 15 s and 60 °C for 45 s. Threshold cycle (Ct) values of assessed miRNAs were compared in samples from different donor groups. The miRNA expression of was evaluated in 2 sets—miR-19b, miR-22, miR-92a, miR-378, miR-425, and cel-miR-39 and miR-30e, miR-31, miR-125b, miR-200b, miR-205, miR-375, and miR-660.

### 4.6. Statistical Analysis

Statistical analysis was carried out with Statistica 6.0 software. Threshold cycle (Ct) values were used to perform ratio-based normalization, effectively evaluating the relative expression of all possible combinations of any two miRNAs in the sample [54,55]. Because miRNA expression was evaluated in 2 sets of 5 and 7 miRNAs, normalization was only used inside of each group. Thus, 31 miRNA ratios were formed from the 12 analyzed miRNAs. For every ratio, Ct difference (dCt) values and the difference of dCt values (ddCt) between every pair of sample types (urine supernatant–urine EVs, urine–blood plasma, and urine EVs–blood plasma) were calculated. For each miRNA ratio, mean dCt, mean ddCt, and their standard deviations were calculated in each of the fractions of biological fluids. Comparisons between groups were done with one-way ANOVA followed by Fisher’s Post-Hoc Test. *p*-values  <  0.05 were considered statistically significant. Benjamini–Hochberg correction (p_adj_) was used to adjust the statistical significance for multiple comparisons. Power analysis was performed, and minimal sample size required to confirm the detected differences was calculated at 95% and 99% significance and 95% statistical power. The specificity and the sensitivity of the analytical systems were assessed using receiving operator characteristic (ROC) curves. The area under ROC curves (AUC) was used as a measure of the diagnostic performance of miRNA ratios. To examine the correlation of miRNA ratio levels with donor characteristics, Spearman’s correlation coefficient was used.

## 5. Conclusions

In the present study, we validated diagnostic potential of cell-free miRNAs—miR-19b, miR-22, miR-92a, miR-378, miR-425, miR-30e, miR-31, miR-125b, miR-200b, miR-205, miR-375, and miR-660—in blood plasma, clarified urine, and urine extracellular vesicles. Different fractions of biological fluids have distinct miRNA expression profiles, and here we demonstrated the great promise of urine vesicles for PCa diagnosis. Those with the highest potential for PCa diagnosis include miR-19b, miR-30e, miR-31, miR-92a, miR-125, miR-200, miR-205, and miR-660 measured in urine EVs. Selected miRNA ratios allow for efficient PCa detection when combined into the following ratios: miR-125b/miR-30e, miR-200/miR-30e, miR-205/miR-30e, miR-31/miR-30e, miR-660/miR-30e, and miR-19b/miR-92a. These results advance our understanding of cancer biology and present one more step towards development of new PCa diagnostics. The next steps are an independent verification of selected miRNA ratios in a sample of at least 35 donors per group to achieve 99% significance and 95% power followed by the development of a diagnostic panel and the differentiation of low-risk and high-risk PCa using miRNA markers.

## Figures and Tables

**Table 1:** The difference of Ct difference (dCt) (ddCt) values for differentially expressed miRNA pairs in the following groups of comparison: prostate cancer (PCa)–healthy male donor (HD), PCa–benign prostatic hyperplasia (BPH), BPH–HD.

miRNA	PCa–HD		PCa–BPH		BPH–HD	
Pairs	UE	U	UE	U	UE	U
22/19b		−1.7 *	3.2 ***		−2.2 **	−1.6 *
19b/92a	−8.2 ***		−8.4 ***	1.5 *		
378a/19b	1.8 **		2.5 ***	−17 *		
425/19b	1.0 **		1.6 ***			
22/92a	−6.7 ***		−4.7 ***			
378a/92a	−5.9 ***		−5.4 ***			
425/92a	−6.7 ***		−63 ***			
22/378a				1.5 *	−1.5 *	−1.5 *
22/425			1.6 ***		−1.6 **	
31/30e	−7.4 ***				−4.8 **	
125b/30e	−6.0 ***		5.6 ***	−1.3 *		
200b/30e	−8.4 ***				−5.3 **	
205/30e	−7.0 ***		−5.8 ***			
375/30e	−5.0 **		−3.5 **			
660/30e	−4.4 ***		−5.9 ***		1.5 *	
125b/31				−3.0 *	4.3 **	
205/31				−0.74 *		
375/31					3.3 *	
660/31					6.3 **	
200b/125b				3.4 *	−4.9 **	
375/125b			2.1 *			
660/125b	1.7 *				2.0 *	
205/200b				−2.8 *	4.1 *	
375/200b	3.3 *				3.8 *	
660/200b				−2.9 *	6.8 *	
660/375			−2.4 *		3.0 *	
Number of differently expressed miRNA pairs	14	1	14	9	15	2

ddCt values with a common directionality of differences with comparisons between PCa and HD are highlighted. *** *p* < 0.001; ** *p* < 0.01; * *p* < 0.05; UE: ddCt in urine extracellular vesicles (EVs); U: ddCt in clarified urine.

**Table 2:** The dddCt values for differentially expressed miRNA pairs in the following groups of comparison: PCa–HD, PCa–BPH, BPH–HD.

miRNA	PCa–HD			PCa–BPH			BPH–HD		
Ratios	UE-U	P-U	UE-P	UE-U	P-U	UE-P	UE-U	P-U	UE-P
22/19b	2.9 ***	−2.3 *		3.4 ***		2.1 *			
19b/92a	−9.2 ***		−8.5 ***	−9.9 ***		−7.5 ***			
378a/19b	3.2 **			4.4 ***					
425/19b	2.8 **			2.8 **					
22/92a	−6.1 ***		−7.3 ***	−6.0 **		−4.8 **			
378a/92a	−5.6 **		−6.0 ***	−5.2 **		−5.4 ***			
425/92a	−6.2 ***		−7.2 ***	−6.5 ***		−6.2 ***			
22/378a			−1.4 *						2.1 *
22/425						1.4 *			−1.5 *
31/30e	−8.0 ***		−9.5 **	−3.9 **					−4.2 *
125b/30e	5.8 ***		−7.5 ***	−4.3 ***		−6.1 ***			
200b/30e	−9.5 ***		−11.8 ***	−5.3 *		−5.0 **			−4.3 ***
205/30e	−6.7 ***		−9.4 ***	−5.1 *		−7.0 *			
375/30e	−3.3 **		−5.9 ***			−4.3 **			
660/30e	−4.4 ***		− 4.0***	−5.4 ***		−5.7 ***			
200b/125b			−4.3 ***						−2.8 **
375/125b	2.4 *								
660/125b			3.5 ***						2.5 **
375/200b			5.9 ***	3.0 **					3.2 **
660/200b	5.1 *		7.8 ***						5.3 ***
660/375	−1.1 *								2.0 *
Number of differently expressed miRNA pairs	16	1	15	13	0	11	0	0	9

dddCt values with a common directionality of differences with comparisons between PCa and HD are highlighted. *** *p* < 0.001; ** *p* < 0.01; * *p* < 0.05; UE-U: dddCt between urine EVs and clarified urine; P-U: dddCt between clarified urine and blood plasma; UE-P: dddCt between urine EVs and plasma.

**Table 3:** Receiving operator characteristic (ROC) curve analysis: PCa vs. (HD+BPH), sensitivity at 100% specificity.

miRNA Ratios	UE	UE-U	UE-P
**19b/92a**	100	100	90
**22/19b**		90	
22/92a	70	60	60
**378a/19b**	80	90	
378a/92a	60	60	60
425/19b	70		
**425/92a**	80	70	90
**125b/30e**	100	100	100
**200b/30e**	100	90	100
**205/30e**	100	80	
**31/30e**	100	100	60
375/30e			80
**660/30e**	100	60	

UE: dCt in urine EVs; UE-U: ddCt between urine EVs and clarified urine; UE-P: ddCt between urine EVs and plasma; ratios with the highest sensitivity are given in bold.

**Table 4:** ROC curve analysis: PCa vs. HD, sensitivity at 100% specificity.

**miRNA Ratios**	**UE**	**U**	**P**	**UE-U**	**P-U**	**UE-P**
**19b/92a**	100			100		90
22/19b		70		90	90	
**22/92a**	80			60		100
**378a/19b**	78			90		
378a/92a	70					
425/19b	67			70	80	
**425/92a**	90			70		90
**125b/30e**	100			100		100
**200b/30e**	100			90		100
**205/30e**	100		60	90		100
**31/30e**	100			100		70
**375/30e**	90					
378a/425	70					
**660/30e**	100					

UE: dCt in urine EVs; U: dCt in clarified urine; P: dCt in plasma; UE-U: ddCt between urine EVs and clarified urine; P-U: ddCt between clarified urine and blood plasma; UE-P: ddCt between urine EVs and plasma. Classifications with highest sensitivity for each miRNA ratio are given in bold.

**Table 5:** ROC curve analysis: PCa vs. BPH, sensitivity at 100% specificity.

miRNA Ratios	UE	U	P	UE-U	P-U	UE-P
**19b/92a**	100	70	70	100		90
**22/19b**	100		70	90		70
22/92a	70			70		60
**378a/19b**	100		60	90		
378a/92a	60			60		60
**425/19b**	100		70			
**425/92a**	80					90
22/425	80					
**125b/30e**	100			100		100
**125b/31**	100				70	
**200b/125b**	100					
**200b/30e**	100			100		100
205/200b		70				
**205/30e**	100			80		
**31/30e**	100			100		60
**375a/200b**				70		100
375a/30e				80		
660/125b				80		
**660/200b**	100	80				
**660/30e**	100	70		100		100
**660/31**	100			80		
**660/375a**			60	90		

UE: dCt in urine EVs; U: dCt in clarified urine; P: dCt in plasma; UE-U: ddCt between urine EVs and clarified urine; P-U: ddCt between clarified urine and blood plasma; UE-P: ddCt between urine EVs and plasma. Classifications with highest sensitivity for each miRNA ratio are given in bold.

**Table 6:** ROC curve analysis: BPH vs. HD, sensitivity at 100% specificity.

miRNA Ratios	UE	U	P	UE-U	P-U	UE-P
22/19b		75				
22/378a	62					
**22/92a**	100					100
22/425	75					
425/92a						62
125b/30e		87				
125b/31		62				
200b/30e		75				87
200b/125b		87				
205/30e			62			
375/205			75			

UE: dCt in urine EVs; U: dCt in clarified urine; P: dCt in plasma; UE-U: ddCt between urine EVs and clarified urine; P-U: ddCt between clarified urine and blood plasma; UE-P: ddCt between urine EVs and plasma. Classifications with highest sensitivity for each miRNA ratio are given in bold.

**Table 7:** Definitely PCa. The percentage of PCa patients divided from the control group (HD+BPH).

miRNA Ratios	UE	UE-U	UE-P	All Fractions
19b/92a	100%	90%	70%	100%
22/92a	70%	60%	60%	70%
378a/92a	60%	60%	60%	60%
425/92a	80%	70%	80%	90%
125/30e	100%	100%	100%	100%
200b/30e			60%	50%
205/30e	60%			60%
375/30e			70%	60%
660/30e	100%	70%	70%	100%
All ratios	100%	100%	100%	100%

UE: dCt in urine EVs; UE-U: ddCt between urine EVs and clarified urine; UE-P: ddCt between urine EVs and plasma.

**Table 8:** Definitely not PCa. The percentage of HD and BPH patients divided from the control group (PCa).

miRNA Ratios	UE	UE-U	UE-P	All Fractions
19b/92a		63%		63%
22/425	58%			58%
378a/425	74%			74%
125b/30e	100%	100%	100%	100%
200b/30e	100%		68%	100%
205/30e	100%	53%	89%	100%
31/30e	100%	100%		100%
375/30e	84%			58%
660/30e	100%	84%	89%	100%
205/31	74%			74%
660/125b			53%	53%
205/200b	53%			53%
660/200b			53%	53%
660/205			53%	63%
All ratios	100%	100%	100%	100%

UE: dCt in urine EVs; UE-U: ddCt between urine EVs and clarified urine; UE-P: ddCt between urine EVs and plasma.

**Table 9:** Definitely ill. The percentage of PCa and BPH patients divided from the control group (HD).

miRNA Ratios	UE	U	UE-U	UE-P	All Fractions
22/19b		50%			50%
22/92a	50%			72%	72%
378a/92a	50%				50%
425/92a	55%			61%	61%
125b/30e	55%		55%	55%	55%
200/b30e				83%	83%
205/30e				66%	66%
660/30e	55%				55%
19b/92a	55%		55%		55%
All ratios	55%	50%	55%	100%	100%

UE: dCt in urine EVs; U: dCt in clarified urine; UE-U: ddCt between urine EVs and clarified urine; UE-P: ddCt between urine EVs and plasma.

**Table 10:** Definitely healthy. The percentage of HD divided from the control group (PCa and BPH patients).

miRNA Ratios	UE	UE-U	UE-P	All Fractions
31/30e	63%			63%
200b/30e	63%		81%	91%
375/31		54%	63%	73%
660/31	63%		72%	91%
200b/125b	63%		63%	91%
660/125b	54%		82%	91%
375/200b	54%		63%	82%
660/200b	63%	63%	91%	91%
All ratios			91%	91%

UE: dCt in urine EVs; U: dCt in clarified urine; UE-U: ddCt between urine EVs and clarified urine; UE-P: ddCt between urine EVs and plasma.

**Table 11:** Overview of the study population.

		PCa	BPH	HD
**Age**	**(Mean ± SD)**	61.9 ± 6.0	72.2 ± 9.7	54.8 ± 3.6
	**Range**	54–71	63–81	50–60
**Total Prostate-Specific Antigen (PSA), ng/mL**		8.2 ± 0.9	6.3 ± 2.1	0.9 ± 0.1
**PSA**	**T_1_N_0_M_0_**	30%	N/A	N/A
	**T_2_N_0_M_0_**	70%		
**Gleason Score**	**5**	10%	N/A	N/A
	**6**	30%		
	**7**	60%		

## Supplementary Materials

The following are available online at https://www.mdpi.com/2075-4418/10/1/38/s1, Table S1: Sequences of primers and probes used for reverse transcription and TaqMan qPCR, Figure S1: Examples of the qPCR curves for following miRNAs: miR-19b, miR-22, miR-92a, miR-378, miR-425 in urine EVs of healthy donor (A) BPH (B) and PCa (C) patients. Figure S2: Examples of the ROC curves for miRNA ratios with 100% specificity while discriminating PCa patients from healthy donors: (A) (100% sensitivity) miR19b/miR92a in urine EVs, (B) (90% sensitivity): miR375/miR30e in urine EVs, C (80% sensitivity) miR22/miR92a in urine EVs, (D) (70% sensitivity) miR378/miR92a in urine EVs, (E) (60% sensitivity) miR205/miR30e in blood plasma, (F) (40% sensitivity) miR125/miR30e in blood plasma. Ratios like miR125/miR30e in blood plasma were considered as low sensitive and are not shown in the manuscript tables.
