# Supplementary material for: The Panel of 12 Cell-Free MicroRNAs as Potential Biomarkers in Prostate Neoplasms
Source: Diagnostics (Basel). 2020 Jan 10;10(1):38. doi: 10.3390/diagnostics10010038 (PMC7168237; doi:10.3390/diagnostics10010038)
Supplement: Supplementary file 1 [file diagnostics-10-00038-s001.pdf]

diagnostics

MDPI

Table 1. Sequences of primers and probes used for reverse transcription and TaqMan qPCR.

miRNA	Label	Sequence
	Universal Reverse Primer	5′-GTGCAGGGTCCGAGGT-3′
**hsa-miR-19b-3p**	RT	5′-GTCGTATCCAGTGCAGGGTCCGAGGTATTCGCACTGGATACGACTCAGTT-3′
	Forward	5′-CGCTGTGCAAATCCATGCAA-3′
	Probe	5′-(FAM)-GCACTGGATACGACTCAGTT-(BHQ)-3′
**hsa-miR-22**	RT	5′-GTCGTATCCAGTGCAGGGTCCGAGGTATTCGCACTGGATACGACACAGTT-3′
	Forward	5′-AAGCTGCCAGTTGAAG-3′
	Probe	5′-GCACTGGATACGACACAGTT-3′
**hsa-miR-92a**	RT	5′-GTCGTATCCAGTGCAGGGTCCGAGGTATTCGCACTGGATACGACACAGGC-3′
	Forward	5′-CGCTATTGCACTTGTCCCG-3′
	Probe	5′-(FAM)-CGCACTGGATACGACACAGGC-(BHQ)-3′
**hsa-miR-378a-3p**	RT	5′-GTCGTATCCAGTGCAGGGTCCGAGGTATTCGCACTGGATACGACGCCTTC-3′
	Forward	5′-GACTGGACTTGGAGTCA-3′
	Probe	5′-(FAM)-CGCACTGGATACGACAAAGTC-(BHQ)-3′
**hsa-miR-425-5p (miR-425)**	RT	5′-GTCGTATCCAGTGCAGGGTCCGAGGTATTCGCACTGGATACGACTCAACG-3′
	Forward	5′-TAATGACACGATCACTCC-3′
	Probe	5′-(FAM)-CGCACTGGATACGACTCAACG-(BHQ)-3′
**hsa-miR-30e**	RT	5′-GTCGTATCCAGTGCAGGGTCCGAGGTATTCGCACTGGATACGACCTTCCA-3′
	Forward	5′-GCCCTGTAAACATCCTTGAC-3′
	Probe	5′-(FAM)-GCACTGGATACGACCTTCCA-(BHQ)-3′
**hsa-miR-31-5p**	RT	5′-GTCGTATCCAGTGCAGGGTCCGAGGTATTCGCACTGGATACGACAGCTAT-3′
	Forward	5′-CAGGCAAGATGCTGGCA-3′
	Probe	5′-(FAM)-TCGCACTGGATACGACAGCTAT-(BHQ)-3′
**hsa-miR-125b-5p**	RT	5′-GTCGTATCCAGTGCAGGGTCCGAGGTATTCGCACTGGATACGACTCACAA-3′
	Forward	5′-CGTCCCTGAGACCCTAACTT-3′
	Probe	5′-(FAM)-GCACTGGATACGACTCACAA-(BHQ)-3′
**hsa-miR-200b-3p**	RT	5′-GTCGTATCCAGTGCAGGGTCCGAGGTATTCGCACTGGATACGACTCATCA-3′
	Forward	5′-GTAATACTGCCTGGTAATG-3′
	Probe	5′-(FAM)-CGCACTGGATACGACTCATCA-(BHQ)-3′
**hsa-miR-205-5p**	RT	5′-GTCGTATCCAGTGCAGGGTCCGAGGTATTCGCACTGGATACGACCAGACT-3′
	Forward	5′-CCTCCTTCATTCCACCGGA-3′
	Probe	5′-(FAM)-GCACTGGATACGACCAGACT-(BHQ)-3′
**hsa-miR-375**	RT	5′-GTCGTATCCAGTGCAGGGTCCGAGGTATTCGCACTGGATACGACTCACGC-3′
	Forward	5'-GCCCTTTGTTCGTTCGGCTC-3'
	Probe	5′-(FAM)-TCGCACTGGATACGACTCACGC-(BHQ)-3′
**hsa-miR-660-5p**	RT	5′-GTCGTATCCAGTGCAGGGTCCGAGGTATTCGCACTGGATACGACCAACTC-3′
	Forward	5′-CCCATTGCATATCGGAG-3′
	Probe	5′-(FAM)-GCACTGGATACGACCAACTC-(BHQ)-3′
**cel-miR-39-3p**	RT	5′-GTCGTATCCAGTGCAGGGTCCGAGGTATTCGCACTGGATACGACCAAGCT-3′
	Forward	5′-ATTCACCGGGTGTAAATC-3′
	Probe	5′-(FAM)-CACTGGATACGACCAAGCTGA-(BHQ)-3′

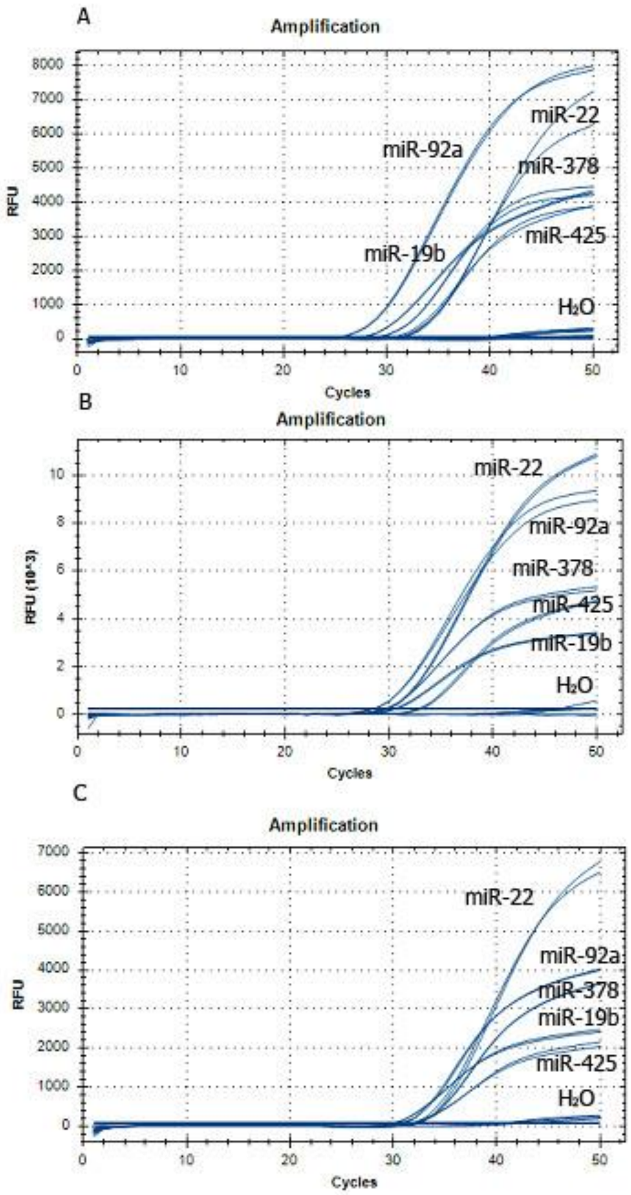

Figure 1. Examples of the qPCR curves for following miRNAs: miR-19b, miR-22, miR-92a, miR-378, miR-425 in urine EVs of healthy donor (A) BPH (B) and PCa (C) patients.

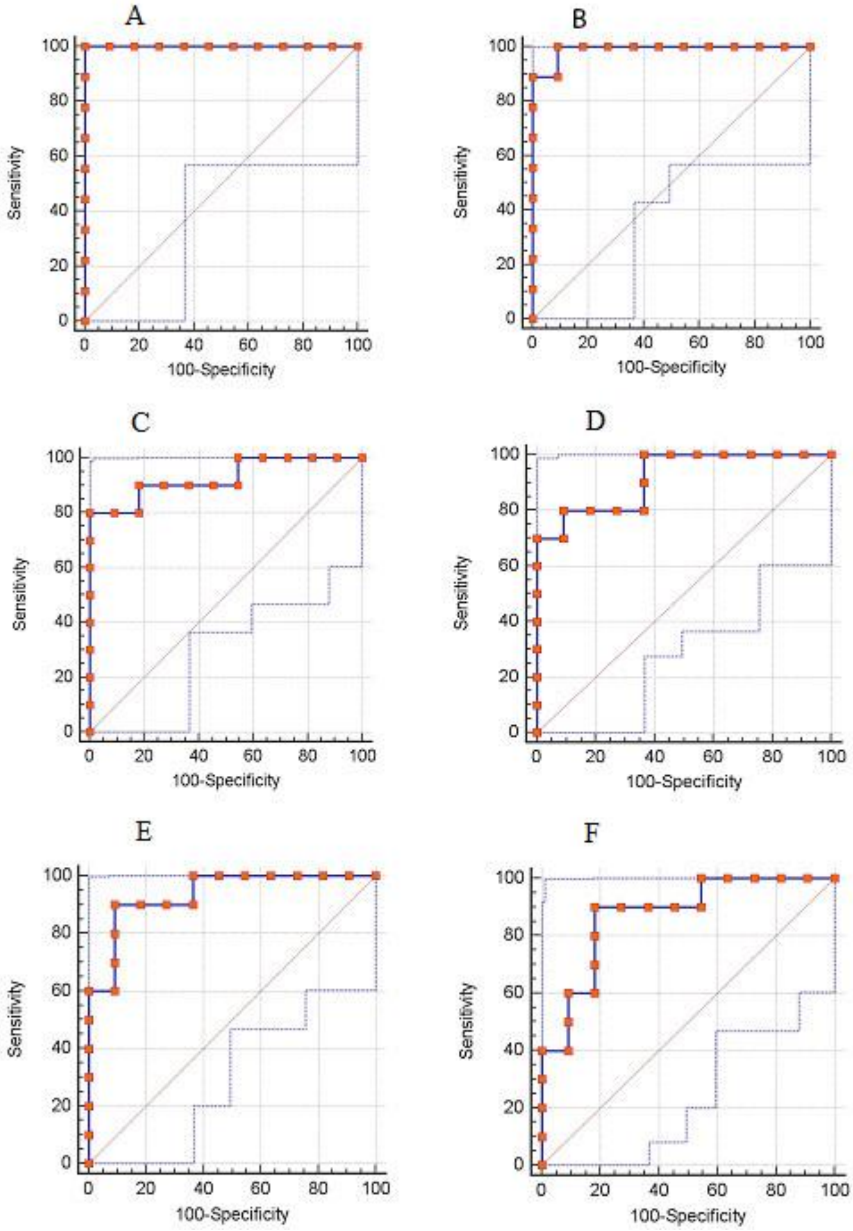

Figure 2. Examples of the ROC curves for miRNA ratios with 100% specificity while discriminating PCa patients from healthy donors: A (100% sensitivity) miR19b / miR92a in urine EVs, B (90% sensitivity): miR375 / miR30e in urine EVs, C (80% sensitivity) miR22 / miR92a in urine EVs, D (70% sensitivity) miR378 / miR92a in urine EVs, E (60% sensitivity) miR205 / miR30e in blood plasma, F (40% sensitivity) miR125 / miR30e in blood plasma. Ratios like miR125 / miR30e in blood plasma were considered as low sensitive and are not shown in the manuscript tables.
